# Parsing stigma's relationship with the psychosocial functioning of youth identified as at clinical high risk for psychosis: evaluating whether symptom stigma or labelling stigma is stronger

**DOI:** 10.1192/bjp.2024.209

**Published:** 2025-05

**Authors:** Lawrence H. Yang, Margaux M. Grivel, Drew Blasco, Ragy R. Girgis, Debbie Huang, Kristen A. Woodberry, Cheryl M. Corcoran, William R. McFarlane, Bruce G. Link

**Affiliations:** Department of Social and Behavioral Sciences, School of Global Public Health, New York University, New York, USA; Department of Social and Behavioral Health, School of Public Health, University of Nevada, Las Vegas, USA; Department of Psychiatry, College of Physicians and Surgeons, Columbia University, New York, USA; New York State Psychiatric Institute, New York, USA; Department of Health Science, California State University, Long Beach, USA; Commonwealth Research Center, Beth Israel Deaconess Medical Center, Boston, USA; Department of Psychiatry, Tufts University, Boston, USA; Department of Psychiatry, Icahn School of Medicine at Mount Sinai, New York, USA; Mental Illness Research, Education, and Clinical Center (MIRECC VISN 2), James J. Peter Veterans Affairs Medical Center, New York, USA; Commonwealth Research Center, Beth Israel Deaconess Medical Center, Boston, USA; Center for Clinical and Translational Science at MaineHealth Institute of Research, Portland, USA; and Department of Psychiatry, Tufts University School of Medicine, Boston, USA; Department of Psychiatry, MaineHealth Institute for Research, Portland, USA; and Department of Psychiatry, Tufts University, Boston, USA; School of Public Policy and Department of Sociology, University of California, Riverside, USA

**Keywords:** Stigma and discrimination, clinical high risk for psychosis, psychotic disorders/schizophrenia, mental health services, stigma interventions

## Abstract

**Background:**

The clinical high risk for psychosis (CHR-p) syndrome enables early identification of individuals at risk of schizophrenia and related disorders. We differentiate between the stigma associated with the at-risk identification itself (‘labelling-related’ stigma) versus stigma attributed to experiencing mental health symptoms (‘symptom-related’ stigma) and examine their relationships with key psychosocial variables.

**Aims:**

We compare labelling- and symptom-related stigma in rates of endorsement and associations with self-esteem, social support loss and quality of life.

**Method:**

We assessed stigma domains of shame-related emotions, secrecy and experienced discrimination for both types of stigma. Individuals at CHR-p were recruited across three sites (*N* = 150); primary analyses included those who endorsed awareness of psychosis risk (*n* = 113). Paired-sample *t*-tests examined differences in labelling- versus symptom-related stigma; regressions examined associations with psychosocial variables, controlling for covariates, including CHR-p symptoms.

**Results:**

Respondents reported greater symptom-related shame, but more labelling-related secrecy. Of the nine significant associations between stigma and psychosocial variables, eight were attributable to symptom-related stigma, even after adjusting for CHR-p symptoms.

**Conclusions:**

Stigma attributed to symptoms had a stronger negative association with psychosocial variables than did labelling-related stigma among individuals recently identified as CHR-p. That secrecy related to the CHR-p designation was greater than its symptom-related counterpart suggests that labelling-related stigma may still be problematic for some CHR-p participants. To optimise this pivotal early intervention effort, interventions should address the holistic ‘stigmatising experience’ of having symptoms, namely any harmful reactions received as well as participants’ socially influenced concerns about what their experiences mean, in addition to the symptoms themselves.

Early identification and intervention for individuals at clinical high risk for psychosis (CHR-p) has provided opportunities to reduce symptoms and potentially prevent transition to threshold psychosis.^1^ Given consensus that individuals with a CHR-p syndrome merit identification and treatment,^1–3^ it is critical to identify how different types of stigma may be associated with psychosocial functioning and recovery.^4–8^ Our research programme proposed that the publicly held conceptions associated with a psychosis risk syndrome (i.e. the CHR-p ‘label’) could exert negative effects, in addition to the symptoms and behaviours that are likely to influence stigma.^9^ Studies of public attitudes show that psychosis-related labels, including CHR-p, as well as symptom manifestations independent of labelling, elicit stigmatising responses such as fear, perceptions of risk for violence and desire for social distance comparable in strength to the diagnosis of ‘schizophrenia’.^10–12^ In light of this, we sought to clarify the comparative contributions of two main types of stigma for individuals identified as CHR-p: (a) ‘labelling-related’ stigma, or stigma associated with the CHR-p label itself and (b) ‘symptom-related’ stigma, or stigma arising from mental health symptoms and experiences in the period preceding, or at the time of, CHR-p identification.^9,13^ In a first single-site study with 38 CHR-p participants,^13^ we found that symptom-related stigma was more salient when compared with labelling-related stigma using our newly developed measures. Further, stigma associated with symptoms was found to be associated with depression, while stigma related to labelling was associated with anxiety. This initial study focused on stigma's associations with symptomatology, thereby gauging the potential relationship of symptoms with the experience of stigma.

## Current study: advances in the assessment of stigma in CHR-p

In the current study, we are interested in the potential stigma correlates of a range of psychosocial variables, controlling for CHR-p symptom severity. In addition, the previous study^13^ included participants whose average attendance in the CHR-p programme was 11.5 months, a period long enough to expect a treatment-initiated abatement of symptoms, which could lead to attenuation of stigma. The current study advances this area by examining, within a multi-site sample of individuals who had been recently (typically <1 month and no more than 6 months) identified as at CHR-p, which type of stigma (label or symptoms) is endorsed more highly and which type shows significant associations with psychosocial factors of self-esteem, social support and quality of life (per prior review^14^). The current study provides another advance by assessing individuals’ awareness of being at risk for psychosis; this is crucial when measuring labelling-related stigma in particular, as up to one-quarter of individuals who meet criteria for CHR-p are not aware of, or do not acknowledge, being at risk for a psychotic disorder which may be, in part, because of the lack of exposure to and understanding of clinical terminology when CHR-p status is communicated.^15,16^

## Study hypotheses and implications

Conveying the at-risk psychosis label can elicit both beneficial and detrimental effects^17,18^; these include relief in learning the identification (e.g. a positive effect^19,20^) and negative expectations of how others would perceive them (e.g. a stigmatising effect^5,21,22^). Alternatively, stigmatising responses can arise in regard to symptoms.^23–25^ The stigma of mental health symptoms encompasses the multifactorial experience of psychosis risk syndromes, and also reflects the experience of independent and overlapping dimensions of symptom development (e.g. depression, anxiety and attentional and other cognitive challenges).^26^ Based upon previous findings showing symptom-related stigma to be more prominent than labelling-related stigma among individuals with CHR-p,^13^ we hypothesise the following: (a) symptom-related stigma will be greater than labelling-related stigma across stigma domains; and (b) symptom-related stigma will show significant associations with psychosocial variables of self-esteem, social support and quality of life when comparative effects of labelling-related stigma are simultaneously accounted for. Identifying the comparative impacts of labelling- versus symptom-related stigma is important in terms of weighing up the benefits and risks in regards to either type of stigma during CHR-p identification,^17,18,27–29^ and for the clinical management of stigma-related issues to reduce its burden among patients.

## Method

Baseline data for 150 help-seeking individuals who met CHR-p criteria was collected from February 2013 to September 2016 via a longitudinal multi-site study conducted in three out-patient clinical high-risk (CHR) clinics at Beth Israel Deaconess Medical Center/Harvard Medical School (Boston, MA), MaineHealth Institute for Research (Portland, ME) and New York State Psychiatric Institute/Columbia University (NYSPI; New York, NY). Participants were referred to mental health treatment if not already receiving it.

### Participants

Participants were help-seeking individuals, aged 12–35 years, who met criteria for one of more of three CHR-p syndromes assessed by the Structured Interview for Psychosis-Risk Syndromes (SIPS, Version 5.0)^30^: brief intermittent psychosis syndrome, attenuated positive symptom syndrome and genetic risk and functional decline syndrome. Exclusion criteria included history of threshold psychosis, acute risk of self-harm/violence, major medical/neurological disorder and IQ < 70. All participants provided written informed consent; minors provided written informed assent with written informed consent from a legal guardian.

All procedures contributing to this work comply with the ethical standards of the relevant national and institutional committees on human experimentation and with the Helsinki Declaration of 1975, as revised in 2013. All procedures involving human participants were approved by the Beth Israel Deaconess Medical Center (IRB# 2016P000183), MaineHealth Institute for Research (IRB# 3996), NYSPI (IRB# 7112R) and New York University (IRB# FY2016-1286) institutional review boards.

### Measures

#### Sociodemographic and clinical covariates

##### Demographics questionnaire

Demographic variables, including age, years of education, gender, country of birth, preferred language, annual household income, marital status, employment status, student enrolment status, race/ethnicity and family history of psychosis, were collected via self-report.

##### Symptoms and functioning

The clinician-administered SIPS assesses positive, negative, disorganised and general symptoms.^30^ Individual items are rated from 0 (absent) to 6 (extreme) and summed to generate symptom scores for positive (five items; range = 0–30), negative (six items; range = 0–36), disorganised (four items; range = 0–24) and general (four items; range = 0–24) symptom subscales. The modified Global Assessment of Functioning (GAF^31^) is a clinician-rated assessment of the impacts of symptoms on daily life (range = 0–100, with higher scores reflecting greater functioning). The Structured Clinical Interview for Diagnostic and Statistical Manual of Mental Disorders, Version IV (SCID IV^32^) was used to identify comorbid Axis I disorders.

##### Awareness of psychosis risk

Per prior work,^33^ participants were asked two questions: (a) ‘Has anyone told you that you were ‘at-risk for’ or ‘developing’ [condition]?’); and (b) ‘Do you think you are at risk for or developing [condition]?’. The five conditions were ‘depression’, *‘*anxiety’, ‘bipolar’, ‘psychosis’ and ‘schizophrenia’. Respondents who endorsed either being ‘told’ or ‘thinking’ they were at risk for ‘psychosis’ or ‘schizophrenia’ were included as being aware of their psychosis risk state. Endorsement of being ‘told’ or ‘thinking’ one was at risk for ‘psychosis’ or ‘schizophrenia’ could arise from their self-perception and/or from being told by others, including receiving a formal CHR-p diagnosis at a CHR programme (below).

#### Independent variables

##### Stigma (labelling-related and symptom-related versions)

Three stigma domains were assessed^13^ – ‘negative emotions’ (referring to shame-related emotions); ‘secrecy’; and ‘experienced discrimination’ (henceforth abbreviated as ‘discrimination’). For each stigma domain, parallel items assessed labelling-related versus symptom-related stigma. Items related to high-risk psychosis labelling stigma assessed experiences of being identified as at CHR-p using the anchor ‘About being told I am at-risk for or developing psychosis … ’. Symptom-related stigma was assessed using the anchor ‘About my symptoms and experiences … ’. To capture the full scope of heterogeneous symptom experiences in this sample, the anchor did not specify the type of symptom. Accordingly, participants may have interpreted this item in terms of a wide range of specific (e.g. perceptual disturbances) and non-specific (e.g. anhedonia) symptoms associated with psychosis, or any comorbid symptoms (e.g. depression, anxiety). All other phrasing was identical across item versions, enabling direct comparison (see Supplementary Table 1 available at https://doi.org/10.1192/bjp.2024.209, including alternate wording for ‘labelling-related’ stigma items if respondents did not report being aware of their psychosis risk). Higher summed scores reflect greater endorsement of stigma.

- Negative emotions.^13^ Three shame-related items (rated 1 [not at all] to 4 [a lot]; range = 3–12) assessed ‘shame’, ‘embarrassment’ or ‘feeling different from others’ attributed to either labelling-related (Cronbach's α = 0.75) or symptom-related (Cronbach's α = 0.80) types.
- Secrecy.^13^ Five items (scored 0 [no] or 1 [yes]; range = 0–5) assessed whom the respondent had told of their CHR-p label (Cronbach's α = 0.72) or symptoms (Cronbach's α = 0.66).
- Discrimination.^13^ Five items (rated 1 [never] to 5 [very often]; range = 5–25) assessed experienced unfair treatment from others attributed to either labelling-related (Cronbach's α = 0.90) or symptom-related (Cronbach's α = 0.92) types.

#### Dependent variables

##### Rosenberg Self-esteem Scale

The Rosenberg Self-esteem Scale^34^ has 10 items (scored 1 [strongly agree] to 4 [strongly disagree]) assessing global self-worth (Cronbach's α = 0.90; range = 10–40).

#### Word Health Organization Quality of Life Scale-Brief

To confirm factor structure and reduce the number of outcomes, the World Health Organization Quality of Life Scale-Brief 26-item measure (items rated 1–5) was factor analysed.^35,36^ Based on examination of the scree plot and confirmed by parallel analysis, a three-factor solution was implemented using Direct Oblimin rotation. The three factors accounted for 49.6% of variability (Supplementary Table 2) and were treated as dependent variables. Subscale scores were generated for the following: (a) ‘satisfaction with life and functioning’ (nine items; range = 9–45; Cronbach's α = 0.83); (b) ‘satisfaction with environment’ (eight items; range = 8–40; Cronbach's α = 0.80); and (c) ‘satisfaction with relationships’ (three items; range = 3–15; Cronbach's α = 0.75). The single-item ‘satisfaction with health’ comprised a final subscale (range = 1–5), as per the original scale, and it remained separate from extracted factors.

##### Norbeck Social Support Questionnaire

We used the Norbeck Social Support Questionnaire (NSSQ)^37^ to assess loss of social support by summing (scored 0 [no], 1 [yes]; range = 0–3) participants’ loss of important relationships owing to ‘others finding out about’, ‘feeling different from others due to’ and ‘not wanting to burden others about’ their mental health condition or treatment (Cronbach's α = 0.64).

### Data analysis

Primary analyses are reported for participants with complete data who reported any awareness of psychosis risk (*n* = 113), as this group could most adequately reflect upon ‘labelling-related’ stigma for CHR-p (supplementary analyses for two additional subsamples were conducted to corroborate findings with this primary analytic group, below). Characteristics were summarised using descriptive statistics (Table 1). For each stigma domain (shame-related emotions, secrecy, discrimination), a paired-sample *t*-test assessed differences between labelling- versus symptom-related subscales.

**Table 1:** Sample characteristics for individuals at clinical high risk for psychosis who endorsed awareness of psychosis risk, *N* = 113

Variable	*N*	*M* (s.d.)
Age (years)	112	18.6 (3.7)
Years of education	111	12.0 (2.9)
Symptoms^a^		
Total positive	113	14.2 (3.6)
Total negative	107	15.3 (6.5)
Total disorganised	107	7.4 (3.9)
Total general	107	11.5 (4.1)
Current GAF^b^	112	46.4 (10.0)
		***n* (%)**
Gender: male	111	70 (63.1%)
Recruitment site	113	
Boston		48 (42.5%)
Maine		37 (32.7%)
New York		28 (24.8%)
Born in the USA	111	104 (93.7%)
Preferred language: English	111	108 (97.3%)
Income (dollars/year)^c^	111	
Less than US$19 999		16 (14.4%)
US$20 000–59 999		17 (15.3%)
Greater than US$59 999		35 (31.5%)
Comorbid Axis I symptoms		
Depression/MDD	95	63 (66.3%)
Anxiety disorders	96	57 (59.4%)
ADHD	95	15 (15.8%)
Marital status: not married	111	108 (97.3%)
Currently employed (full/part)	111	35 (31.5%)
Enrolled as a student	111	91 (82.0%)
Race/ethnicity	111	
Hispanic		15 (13.5%)
Non-Hispanic		96 (86.5%)
White		70 (63.1%)
Black		13 (11.7%)
Asian		6 (5.4%)
Inter-racial		5 (4.5%)
Other		2 (1.8%)
Self-reported family history of psychosis: present	111	39 (35.1%)

GAF, Global Assessment of Functioning; ADHD, attention-deficit hyperactivity disorder; MDD, major depressive disorder.

a. Ranges for symptom subscales are 0–30 (positive symptoms), 0–36 (negative symptoms) and 0–24 (disorganised and general symptoms).

b. Range for the GAF is 0–100.

c. Column percentage does not sum to 100% as 43 (38.7%) respondents reported ‘not knowing’.

Next, we examined associations between stigma type and dependent variables. We first conducted separate multivariate linear regressions for each stigma domain to test: (a) the independent association of labelling- and symptom-related stigma with the joint distribution of dependent variables; and (b) the combined association of labelling- and symptom-related stigma with the joint distribution of dependent variables, with and without adjusting for covariates (site, age, gender, race/ethnicity, family history of psychosis and positive, negative and disorganised symptoms;^33^ Supplementary Table 3).

Next, bivariate models were used to independently assess the association of the labelling- and symptom-related version of each stigma domain with psychosocial variables (Table 2), while multivariable models were used to associate labelling- and symptom-related stigma domains simultaneously (i.e. controlling for the effect of the other) with each of the psychosocial variables (Table 3) (henceforth, ‘multivariable model’). Based on which stigma type remained significant (*P* < 0.05), a predominant stigma type was identified for use in the ‘final adjusted models’ that were adjusted for covariates (demographics and symptoms) (Table 3; Supplementary Table 4 shows parameter estimates for all variables). Bivariate, multivariable and final adjusted modelling was also conducted for the entire sample, including all participants who met CHR-p criteria, regardless of awareness of psychosis risk (*n* = 150; Supplementary Tables 5 and 6), and for the subset of individuals who reported awareness of psychosis risk and who received a formal CHR-p diagnosis at a CHR programme (*n* = 89; Supplementary Tables 7 and 8) (see Supplementary Figure 1 for flowchart of subsamples). Lastly, exploratory interaction analyses were conducted to examine the implications of race/ethnicity^38^ and family history of psychosis^39^ on associations between stigma and psychosocial variables (Supplementary Tables 9 and 10), as these have shown salience in relation to mental illness stigma. Significant interactions are cautiously presented in these supplementary tables given the likelihood of Type 1 error.

**Table 2:** Bivariate models between stigma domain (shame, secrecy and discrimination) and psychosocial variables: linear regression results, *N* = 113

Type of stigma	Self-esteem	Social support loss	QOL: satisfaction with life and functioning	QOL: satisfaction with environment	QOL: satisfaction personal relationships	QOL: satisfaction with health
	*B* (95% CI)	*B* (95% CI)	*B* (95% CI)	*B* (95% CI)	*B* (95% CI)	*B* (95% CI)
*Panel A: shame-related emotions*						
*N*	92	99	96	96	96	96
Labelling	−0.76** **(−1.30, −0.22)**	0.16*** **(0.10, 0.23)**	−0.05 (−0.11, 0.00)	−0.05 (−0.11, 0.01)	−0.07 (−0.14, 0.01)	−0.04 (−0.1, 0.05)
Symptom	−1.11*** **(−1.63, −0.59)**	0.17*** **(0.11, 0.24)**	−0.11*** **(−0.16, −0.06)**	−0.06* **(−0.12, −0.01)**	−0.07 (−0.15, 0.01)	−0.13** **(−0.21, −0.04)**
*Panel B: secrecy*						
*N*	91	97	94	94	94	94
Labelling	0.20 (−0.64, 1.04)	0.07 (−0.04, 0.19)	0.05 (−0.04, 0.13)	0.04 (−0.04, 0.13)	−0.10 (−0.22, 0.01)	0.12 (−0.01, 0.25)
Symptom	0.24 (−0.69,1.16)	−0.01 (−0.14, 0.12)	0.06 (−0.04, 0.15)	0.01 (−0.09, 0.11)	−0.04 (−0.17, 0.10)	0.23** **(0.09, 0.37)**
*Panel C: discrimination*						
*N*	91	98	95	95	95	95
Labelling	−0.41** **(−0.66, −0.17)**	0.02 (−0.01, 0.06)	−0.03* **(−0.05, −0.00)**	−0.01 (−0.04, 0.02)	−0.02 (−0.06, 0.01)	−0.03 (−0.07, 0.01)
Symptom	−0.39** **(−0.66, −0.11)**	0.10*** **(0.07, 0.13)**	−0.05*** **(−0.08, −0.02)**	−0.05*** **(−0.08, −0.02)**	−0.06** **(−0.10, −0.02)**	−0.06** **(−0.10, −0.02)**

QOL, quality of life.

Betas are unstandardised. Significance at *P < 0.05, **P < 0.01, ***P < 0.001.

**Table 3:** Linear regression models between stigma domain (shame, secrecy and discrimination) and psychosocial variables: multivariable models and adjusted models, *N* = 113

Type of stigma	Self-esteem	Social support loss	QOL: satisfaction with life and functioning	QOL: satisfaction with environment	QOL: satisfaction personal relationships	QOL: satisfaction with health
	*B* (95% CI)	*B* (95% CI)	*B* (95% CI)	*B* (95% CI)	*B* (95% CI)	*B* (95% CI)
*Panel A: shame-related emotions*						
*N*	92	99	96	96	96	96
Multivariable model						
Labelling	−0.05 (−0.74, 0.65)	0.08 (−0.01, 0.17)	0.04 (−0.03, 0.11)	−0.01 (−0.09, 0.07)	−0.03 (−0.14, 0.07)	0.10 (−0.01, 0.21)
Symptom	−1.08** **(−1.78, −0.38)**	0.12* **(0.03, 0.21)**	−0.14*** **(−0.21, −0.06)**	−0.05 (−0.13, 0.03)	−0.05 (−0.15, 0.06)	−0.19*** **(−0.31, −0.08)**
Final adjusted model						
*Final subscale:*	*Symptom*	*Symptom*	*Symptoms*	–	–	*Symptoms*
	−1.20*** **(−1.72, −0.68)**	0.16*** **(0.09, 0.23)**	−0.10*** **(−0.15, −0.04)**	–	–	−0.12* **(−0.21, −0.03)**
*Panel B: secrecy*						
*N*	91	97	94	94	94	94
Multivariable model						
Labelling	0.12 (−0.87, 1.12)	0.11 (−0.02, 0.24)	0.03 (−0.07, 0.13)	0.06 (−0.05, 0.16)	−0.12 (−0.26, 0.02)	0.02 (−0.13, 0.16)
Symptom	0.17 (−0.93, 1.26)	−0.07 (−0.22, 0.07)	0.04 (−0.07, 0.15)	−0.02 (−0.14, 0.09)	0.04 (−0.12, 0.19)	0.22** **(0.06, 0.38)**
Final adjusted model						
*Final subscale:*	–	–	–	–	–	*Symptoms*
	–	–	–	–	–	0.23** **(0.09, 0.38)**
*Panel C: discrimination*						
*N*	91	98	95	95	95	95
Multivariable model						
Labelling	−0.31* **(−0.60, −0.02)**	−0.03 (−0.06, 0.01)	0.01 (−0.04, 0.02)	0.02 (−0.01, 0.05)	0.00 (−0.04, 0.04)	0.00 (−0.05, 0.04)
Symptom	−0.21 (−0.52, 0.10)	0.11*** **(0.08, 0.15)**	−0.04** **(−0.07, −0.01)**	−0.06*** **(−0.09, −0.03)**	−0.06** **(−0.10, −0.02)**	−0.06* **(−0.11, −0.01)**
Final adjusted model						
*Final subscale:*	*Labelling*	*Symptom*	*Symptoms*	*Symptoms*	*Symptoms*	*Symptoms*
	−0.37** **(−0.62, −0.11)**	0.10*** **(0.07, 0.13)**	−0.04** **(−0.07, −0.01)**	−0.04** **(−0.07, −0.02)**	−0.0 (−0.07, 0.01)	−0.04 (−0.08, 0.01)

QOL, quality of life.

Betas are unstandardised. Significance at *P < 0.05, **P < 0.01, ***P < 0.001. Final adjusted models adjust for: age, gender, race/ethnicity, family history of psychosis, total positive symptoms, total negative symptoms, total disorganised symptoms and site; double-dash (–) in unpopulated cells indicates that neither the labelling nor the symptom subscale was moved to the final adjusted model.

Statistical significance was considered *P* < 0.05. Analyses were cross-checked by two independent teams using SPSS version 26/27 and SAS version 9.4 (Cary, NC, USA; see https://www.sas.com/en_us/home.html).

## Results

### Awareness of psychosis risk

For the primary analyses, 113 participants endorsed having been told or thinking they were at risk for ‘psychosis’ or ‘schizophrenia’.

### Sample characteristics

Participants were primarily students (82%), male (63.1%) and White (63.1%). Participants had a mean age of 18.6 years (s.d. = 3.7), reported moderate positive (*M* = 14.2; s.d. = 3.6), negative (*M* = 15.3; s.d. = 6.5) and general (*M* = 11.5; s.d. = 4.1) symptoms, reported some disorganised symptoms (*M* = 7.4; s.d. = 3.9) and reported marked functional impairment (GAF *M* = 46.4; s.d. = 10.0) (Table 1).

### Is stigma higher based on one's label or one's symptoms?

Participants endorsed greater symptom-related (*M* = 7.0; s.d. = 2.3) versus labelling-related (*M* = 6.3; s.d. = 2.3) shame (*t*(109) = 3.71, *P* < 0.001). Conversely, participants endorsed greater labelling-related (*M* = 2.7; s.d. = 1.6) versus symptom-related (*M* = 1.9; s.d. = 1.4) secrecy (*t*(106) = 5.37, *P* < 0.001). There was no significant difference in endorsement of symptom-related (*M* = 10.0; s.d. = 4.8) versus labelling-related (*M* = 9.7; s.d. = 5.0) discrimination (*t*(108) = 0.67, *P* = 0.51).

### What is the overall relationship of stigma type to all dependent variables?

Multivariate omnibus tests of each stigma type on the joint distribution of dependent variables show that symptom stigma appears more strongly associated (η^2^) than labelling stigma across all dependent variables, even after accounting for covariates (η^2^ = 0.11–0.35 for symptom-related stigma domains versus η^2^ = 0.06–0.17 for labelling-related stigma domains) (Supplementary Table 3).

Table 2 shows that, of the 16 statistically significant bivariate relationships between stigma domains and dependent variables, 12 (75%) are attributable to symptom-related stigma and four (25%) to stigma related to the high-risk psychosis label. While both labelling-related and symptom-related stigma domains were associated with self-esteem and social support loss, symptom-related stigma showed eight statistically significant associations with quality-of-life factors whereas labelling-related stigma showed only one statistically significant association.

### Is labelling-related or symptom-related stigma significantly associated with psychosocial factors?

In the following, we describe the multivariable and final adjusted models, focusing on describing associations between labelling-related stigma domains and dependent variables, and symptom-related stigma domains and dependent variables (Table 3).

#### Labelling-related stigma

In the multivariable models, after controlling for symptom-related counterparts, only labelling-related discrimination was significantly associated with lower self-esteem (Table 3). This association remained significant in the final adjusted model (*B* = −0.37; 95% CI [−0.62, −0.11], *R*^2^ = 0.27). Labelling-related emotions and labelling-related secrecy were not significantly associated with any dependent variables in the multivariable model step (Table 3).

Accordingly, when examining the final adjusted models between all stigma domains related to the high-risk psychosis label and dependent variables, only one statistically significant association was found between labelling-related discrimination and self-esteem (Table 4).

**Table 4:** Stigma and psychosocial variables: summary of final adjusted models, *N* = 113

Type of stigma	Self-esteem	Social support loss	QOL: satisfaction with life and functioning	QOL: satisfaction with environment	QOL: satisfaction personal relationships	QOL: satisfaction with health
*Panel A: shame-related emotions*						
Labelling	–	–	–	–	–	–
Symptom	*P* < 0.001 (−)	*P* < 0.001(+)	*P* < 0.001 (−)	–	–	*P* < 0.05(−)
*Panel B: secrecy*						
Labelling	–	–	–	–	–	–
Symptom	–	–	–	–	–	*P* < 0.01 (+)
*Panel C: discrimination*						
Labelling	*P* < 0.01 (−)	–	–	–	–	–
Symptom	–	*P* < 0.001(+)	*P* < 0.01 (−)	*P* < 0.01 (−)	–	–

QOL, quality of life.

In unpopulated cells, a double dash (–) indicates this stigma variable did not advance to the final adjusted model. In populated cells, (−) indicates an inverse relationship between stigma variable and psychosocial variable; (+) indicates a positive relationship between stigma variable and psychosocial variable.

#### Symptom-related stigma

Symptom-related emotions, after controlling for its labelling-related counterpart in the multivariable model, was significantly associated with worse self-esteem, satisfaction with life and functioning, satisfaction with health and greater social support loss (Table 3). These associations remained significant in the final adjusted model for the following: self-esteem (*B* = −1.08; 95% CI [−1.78, −0.38], *R*^2^ = 0.36); satisfaction with life and functioning (*B* = −0.14; 95% CI [−0.21, −0.06], *R*^2^ = 0.31); satisfaction with health (*B* = −0.12; 95% CI [−0.21, −0.03], *R*^2^ = 0.16); and social support loss (*B* = 0.16; 95% CI [0.09, 0.23], *R*^2^ = 0.21) (Table 3).

Symptom-related secrecy, after controlling for its labelling-related counterpart in the multivariable model, was significantly, and somewhat unexpectedly, associated with *greater* satisfaction with health. This association remained significant in the final adjusted model (*B* = 0.23, 95% CI [0.09, 0.38], *R*^2^ = 0.21) (Table 3).

Symptom-related discrimination, after controlling for its labelling-related counterpart in the multivariable model, was significantly associated with reduced satisfaction with life and functioning, satisfaction with environment, satisfaction with personal relationships, satisfaction with health and greater social support loss (Table 3). These associations remained significant in the final adjusted model for the following: satisfaction with life and functioning (*B* = −0.04; 95% CI [−0.07, −0.01], *R*^2^ = 0.26), satisfaction with environment (*B* = −0.04; 95% CI [−0.07, −0.02], *R*^2^ = 0.21) and greater social support loss (*B* = 0.10; 95% CI [0.07, 0.13], *R*^2^ = 0.33) (Table 3).

#### Overall findings

When examining the final adjusted models between all symptom-related stigma domains and dependent variables, eight statistically significant associations were found (Table 4). These associations persisted even after accounting for participants’ positive, negative and disorganised symptoms, with these symptoms remaining significantly associated with various dependent variables in the final models (Supplementary Table 4). Among the symptom-related stigma findings, shame-related emotions (four significant associations) and discrimination (three significant associations) most frequently accounted for the overall relationship between stigma and psychosocial factors.

As an additional check to examine the role of positive symptoms, we explored whether individuals at CHR-p, stratified into ‘high’ versus ‘low’ positive symptoms (per median split), showed differing associations between symptom-related stigma and dependent variables; the results (available upon request) were relatively consistent among subgroups.

### Is this overall pattern corroborated in supplementary CHR-p subsamples?

The bivariate models, multivariable models and final adjusted models were replicated among the following: (a) all individuals who met criteria for CHR-p (*n* = 150; Supplementary Tables 5 and 6) and (b) among individuals reporting awareness of psychosis risk and who were formally conveyed a CHR-p designation (89/113; Supplementary Tables 7 and 8). While isolated variations in significant associations were found, the overall pattern in the final adjusted models remained consistent across the primary sample and these two supplementary samples, corroborating the predominant pattern between symptom-related stigma and dependent variables (i.e. accounting for ten of ten total significant associations in the first supplementary sample [*n* = 150] and seven of eight [87.5%] total significant associations in the second supplementary sample [*n* = 89]).

### Exploration of whether patterns of association differ by race/ethnicity or family history of psychosis

For the primary analyses, interaction analyses were conducted to explore whether race/ethnicity and family history of psychosis affected associations between stigma and psychosocial variables. We tested 22 interactions and found only three (14%) to be significant and none to be large (see Supplementary Tables 9 and 10). In fact, the adjusted *R*-square increment achieved when interaction terms were added was generally very small (mean change = 0.8%; range: −0.33%, 1.47%). As a result, we have chosen not to strongly interpret interactions, and instead include results in the supplement as exploratory findings for future research.

## Discussion

We found that symptom-related stigma had a stronger negative association with psychosocial functioning than did labelling-related stigma in individuals who had been recently identified as meeting criteria for CHR-p and who acknowledged psychosis risk. The finding that associations between stigma and psychosocial variables remain when symptoms are controlled indicates that stigma processes (especially CHR participants’ stigmatising perceptions about symptoms^13^) are an independent factor related to psychosocial outcomes. Stigma related to having symptoms requires addressing in its own right. This is especially true given the critical developmental period during which CHR-p individuals are identified.^9^ Symptom-induced experiences of stigma, especially shame and perceived discrimination, are occurring when identity and social networks are being consolidated^40^ and their negative impacts at this time are reflected in loss of social support and worse quality of life. The comparatively prominent role of symptom-related stigma was corroborated in two supplementary samples: when we included individuals who met CHR-p criteria but who did not acknowledge being at psychosis risk,^33^ and among only those who had both been informed of and acknowledged psychosis risk.

That the stigma reported was primarily associated with symptoms makes sense given the myriad symptoms experienced by individuals identified as at CHR-p.^3,26,30^ CHR-p samples are known to have a range of both independent and overlapping psychotic and non-psychotic symptoms.^26^ Most study participants (>66%) reported comorbid depressive and/or anxiety disorders. As a first step to address symptom-related stigma, community-level campaigns^41^ to help contextualise psychotic-like symptoms could be implemented to reduce community stigma associated with CHR-p symptomatology. In addition to improving lay recognition of the warning signs of early psychosis,^42,43^ such community-level efforts (e.g. in schools and churches) could lower reticence to contact CHR-p programmes among symptomatic individuals by decreasing symptom-related stigma. Community-level campaigns could emphasise accurate information about the risk for transition to threshold psychosis (~25% at 3 years^44^) when CHR-p symptoms are present, which was found to reduce social distancing attitudes among young adults.^11^ Second, future interventions could address stigmatising perceptions associated with symptoms and related experiences by targeting both internalised and external exposures to stigma.^45^ Internalised beliefs about stigma associated with CHR-p and non-CHR-p specific symptoms could manifest in shame, alienation and differentness, or as feelings of ‘not fitting in with others’.^25^ External manifestations of symptom-related stigma – that is, perceived and/or actual negative treatment from others – include being teased, shunned and categorised as odd by peers or family.^5,12^ Stigma interventions can target shame-related emotions using cognitive behavioural^46^ and family psychoeducational^17,42,43^ approaches and discriminatory experiences using adaptive coping strategies. What is key is to deal with the entire range of stigmatising experiences associated with symptoms, their impacts and the attributions assigned to them.^20^ This includes the harmful reactions people receive as well as their own internal but socially influenced concerns about what their emerging problems mean. One key caveat is to approach secrecy related to symptoms and experiences carefully given its apparent and somewhat unexpected protective relationship with satisfaction with health; secrecy about one's symptoms may be adaptive in many circumstances (e.g. to avert school-based bullying^5,24^). Accordingly, interventions could encourage gradual disclosure^47^ of CHR-p symptoms to social circles that individuals identify as safe. Support from peers^48^ with lived experience of CHR-p could help address perceptions of differentness associated with symptoms and in coping with discriminatory experiences.

We found that, after adjusting for symptom-related stigma, only one stigma variable (i.e. discrimination) related to the high-risk psychosis label was associated with psychosocial outcomes (i.e. self-esteem) among our recently identified CHR-p sample. This finding is consistent with our prior study showing that despite mitigating negative emotions (e.g. shame), the communication of CHR-p status by a CHR programme had a negative impact on how individuals viewed themselves and expected others to view them.^20^ Of note, in contrast to our symptom-related stigma probe, our labelling-related stigma probe asked specifically about stigma associated with the high-risk psychosis label. It thus did not encompass other labels (e.g. ‘depressed’, ‘anxious’, ‘weird’, ‘odd’) that could be more relevant to identity, stigma and psychosocial functioning.^5,12,24,25^ Two study findings suggest that labelling-related stigma may still be problematic for a substantial subsample of CHR-p participants. First, approximately one-quarter of our total CHR-p sample (37/150; 24.7%) did not identify with or agree with being at risk for developing schizophrenia or psychosis. Second, we found that secrecy associated with receiving the CHR-p designation was endorsed significantly more highly than secrecy related to symptoms. In either case, respondents may anticipate that community members are likely to conflate ‘psychosis risk’ with the concept of ‘psychosis’, thus evoking comparably severe stereotypes (e.g. of violence) and discrimination (e.g. social distance) from others.^11^ These findings show that many CHR-p participants do not identify with the CHR-p label (whether via lack of exposure to, understanding of^15,16^ or active rejection of the label) and as a result withhold the information from others.

Limitations include the cross-sectional design, which precluded evaluating causality. Second, given recent receipt of the CHR-p designation among study participants, experiences of labelling-related stigma were time-limited. On average, participants were typically assessed at <1 month and no more than 6 months from CHR-p identification, when formal CHR-p labelling is most likely to occur. Accordingly, longitudinal evaluation is warranted to examine how labelling-related stigma could affect psychosocial outcomes over time. Further, individuals who entered specialised CHR-p services could be less susceptible to, and less likely to endorse, stigma associated with the high-risk psychosis label. Labelling-related stigma could also have been underestimated by the focus on the psychosis risk label specifically, as no standardised or uniform guidelines of conveying CHR-p status currently exist^17,18,33^; instead, our findings reflect (and adjust for) the varied and individualised communication methods applied by CHR-p programme clinicians by site. Another limitation is that symptom-related items likely encompassed stigmatising perceptions of co-occurring symptoms and experiences at time of CHR-p identification in addition to those specifically leading to identification. Asking specifically about stigma related to positive (and perhaps negative) symptoms associated with CHR-p might yield different results.

### Future directions

Our findings point to, within a relatively large, multi-site sample, the strong relationship between symptom-related stigma and psychosocial functioning. That identification as CHR-p may have less to do with key aspects of youths’ self-esteem, quality of life and social support than the stigma they associate with the symptoms they experience, independent of the actual symptoms they experience, places the onus on addressing both community-level and internalised stigma in addition to symptoms. We recommend that specialised programmes and adjunctive stigma interventions address perceptions of how people may respond negatively to symptoms and experiences related to CHR-p and how patient-related factors (e.g. cultural meanings related to mental health symptoms;^38^ family history of psychosis^39^) affect individuals; such interventions could take place as community-level campaigns^36,42,43^ and at the level of individual CHR-p participants. We also recommend that future investigations, including qualitative studies,^49^ examine the comparative stigma of different types of labels that these young people may have received or identify with, some of which may be related to the experience of CHR-p, with others relating to the multitude of other factors that come to define individuals as they grow up. Similarly, future studies should examine how specific symptom-related stigma is associated with psychotic versus non-psychotic symptoms, and the processes by which stigma is internalised and subsequently mitigated. In closing, our findings indicate that additional addressing of stigma could maximise youths’ potential for recovery and optimise this pivotal early intervention effort in psychiatry.

## Supporting information

Yang et al. supplementary material Yang et al. supplementary material

## Data Availability

The data that support the findings of this study are available from the corresponding author, L.H.Y., upon reasonable request.

## Analytic code availability

The analytic code used to generate the findings of this study are available from the corresponding author, L.H.Y., upon reasonable request.

## Research material availability

No additional research materials other than de-identified data and analytic code will be made available.
