# Supplementary material for: Parsing stigma's relationship with the psychosocial functioning of youth identified as at clinical high risk for psychosis: evaluating whether symptom stigma or labelling stigma is stronger
Source: Br J Psychiatry. 2024 Dec 4;226(5):288–96. doi: 10.1192/bjp.2024.209 (PMC12116217; doi:10.1192/bjp.2024.209)
Supplement: Yang et al. supplementary material [file S0007125024002095sup001.docx]

| **Supplemental Table 1. Item Wording for Labeling-related and Symptom-related Stigma Items.** | | |
| --- | --- | --- |
| **Labeling-related Stigma Items** | **Symptom-related Stigma Items** | **Response options** |
| ***Panel A: Shame-Related Emotions*** | | |
| **Item Root:** About being told I am at-risk for or developing [X]^a^, I have felt ______. | **Item Root:** About my symptoms and experiences, I have felt ______. | Not at all [1] A little [2] Moderately [3]  A lot [4] |
| 1. …embarrassed | …embarrassed |  |
| 2. …different from others | …different from others |  |
| 3. …ashamed | …ashamed |  |
| ***Panel B: Secrecy*** | | |
| 1. I have told no one that I am at-risk for or developing [X]^a^ | I have told no one that I have symptoms. | Yes [1] No [0]  # ^b^ |
| 2. I have told friends that I am at-risk for or developing [X]^a^ | I have told friends that I have symptoms |  |
| 3. I have told family members that I live with that I am at-risk for or developing [X]^a^ | I have told family members that I live with that I have symptoms |  |
| 4. I have told family members that I don’t live with that that I am at-risk for or developing [X]^a^ | I have told family members that I don’t live with that I have symptoms |  |
| 5. I have told other people besides friends and family that I am at-risk for or developing [X]^a^ | I have told other people besides friends and family that I have symptoms |  |
| ***Panel C: Discrimination*** | | |
| **Item Root:** Because I am at-risk for or developing [X]^a^, people _____ | **Item Root:** Because I have had symptoms, people _____ | Never [1] Seldom [2] Sometimes [3] Often [4] Very often [5] |
| 1. …have treated me differently | …have treated me differently. |  |
| 2. …seem to be less comfortable with me. | …seem to be less comfortable with me. |  |
| 3. …hang out with me less | …hang out with me less. |  |
| 4. …are unfair to me | …are unfair to me. |  |
| 5. …are a little afraid of me | …are a little afraid of me. |  |
| *Note*. ^a^ For analyses with the primary analytic group, “[X]” represents psychosis or schizophrenia. ^b^ In addition to provide a “Yes”/“No” response, participants are asked to indicate the number of people told for Panel B, labeling and symptom items 2–5. | | |

| **Supplemental Table 2.**  **Factor Loadings of WHO Quality of Life Scale Items, *N*=113** | | | | | | |
| --- | --- | --- | --- | --- | --- | --- |
| **Final Factor Names** | **Scale Item #** | **WHO Quality of Life Items** | **Factor Loadings** | | | |
|  |  |  | 1 | 2 | 3 | |
| QOL: Satisfaction with Life & Functioning | 1 | How would you rate your quality of life | **0.611** | -- | -- | |
|  | 5 | How much do you enjoy life? | **0.773** | -- | -- | |
|  | 6 | To what extent do you feel your life to be meaningful? | **0.795** | -- | | -- |
|  | 7 | How well are you able to concentrate? | **0.511** | -- | -- | |
|  | 11 | Are you able to accept your bodily appearance? | **0.526** | -- | -- | |
|  | 16 | How satisfied are you with your sleep? | **0.477** | -- | -- | |
|  | 17 | How satisfied are you with your ability to perform daily activities? | **0.514** | -- | -- | |
|  | 19 | How satisfied are you with yourself? | **0.838** | -- | -- | |
|  | 26 | How often do you have negative feelings such as blue mood | **0.693** | -- | -- | |
|  |  |  |  |  |  | |
| QOL: Satisfaction with Environment | 8 | How safe do you feel in your daily life? | -- | **0.41** | -- | |
|  | 9 | How healthy is your physical environment? | -- | **0.751** | -- | |
|  | 12 | Have you enough money to meet your needs? | -- | **0.557** | -- | |
|  | 14 | To what extent do you have the opportunity for leisure activities? | -- | **0.486** | -- | |
|  | 15 | How well are you able to get around? | -- | **0.625** | -- | |
|  | 23 | How satisfied are you with the conditions of your living place? | -- | **0.85** | -- | |
|  | 24 | How satisfied are you with your access to health services? | -- | **0.622** | -- | |
|  | 25 | How satisfied are you with your transport? | -- | **0.754** | -- | |
|  |  |  |  |  |  | |
| QOL: Satisfaction with Personal Relationships | 20 | How satisfied are you with your personal relationships? | -- | -- | **-0.722** | |
|  | 21 | How satisfied are you with your sex life? | -- | -- | **-0.696** | |
|  | 22 | How satisfied are you with the support you get from your friends? | -- | -- | **-0.739** | |
| ***Note.*** Factor loadings below 0.4 are suppressed (--). Items 10, 13, 18, and 4 were removed from the final analysis as they do not load on any factor. Item 2 also did not load on any of the three factors; however, it was retained as a separate factor, per the original WHO QOL scale as ‘Satisfaction with Health’. Finally, to increase internal consistency and improve conceptual coherence for the domain ‘Satisfaction with Personal Relationships,’ item 3 was removed. | | | | | | |

| **Supplemental Table 3. Overall Effect of Symptom-related Versus Labeling-Related Stigma Type on All Dependent Variables, *N*=113** | | | | | | |  |
| --- | --- | --- | --- | --- | --- | --- | --- |
|  | | ***Wilk’s* L** | **F** | ***df*** | **Error *df*** | **multivariate** η^2^ | |
| ***Panel A: Shame-Related Emotions*** | |  | | | | | |
| Bivariate Model | Labeling | 0.77 | **4.25^***^** | 6 | 84 | 0.23 | |
| Bivariate Model | Symptom | 0.69 | **6.24^***^** | 6 | 84 | 0.31 | |
| Multivariable Model | Labeling | 0.89 | 1.70 | 6 | 83 | 0.11 | |
|  | Symptom | 0.80 | **3.39^**^** | 6 | 83 | 0.20 | |
| Final Adjusted Model | Labeling | 0.88 | 1.73 | 6 | 74 | 0.12 | |
|  | Symptom | 0.81 | **3.00^*^** | 6 | 74 | 0.20 | |
| ***Panel B: Secrecy*** | | | | | | | |
| Bivariate Model | Labeling | 0.91 | 1.44 | 6 | 83 | 0.09 | |
| Bivariate Model | Symptom | 0.87 | 2.00 | 6 | 83 | 0.13 | |
| Multivariable Model | Labeling | 0.93 | 1.03 | 6 | 82 | 0.07 | |
|  | Symptom | 0.90 | 1.57 | 6 | 82 | 0.10 | |
| Final Adjusted Model | Labeling | 0.94 | 0.79 | 6 | 73 | 0.06 | |
|  | Symptom | 0.89 | 1.55 | 6 | 73 | 0.11 | |
| ***Panel C: Discrimination*** | | | | | | | |
| Bivariate Model | Labeling | 0.87 | 2.10 | 6 | 83 | 0.13 | |
| Bivariate Model | Symptom | 0.62 | **8.40^***^** | 6 | 83 | 0.38 | |
| Multivariable Model | Labeling | 0.86 | 2.20 | 6 | 82 | 0.14 | |
|  | Symptom | 0.62 | **8.48^***^** | 6 | 82 | 0.38 | |
| Final Adjusted Model | Labeling | 0.84 | **2.40^*^** | 6 | 73 | 0.17 | |
|  | Symptom | 0.65 | **6.57^***^** | 6 | 73 | 0.35 | |
| *Note.* **^*^p<0.05, ^**^p<0.01, ^***^p<0.001**; Final Adjusted Model adjusts for: age, sex, race/ethnicity, family history of psychosis, total positive symptoms, total negative symptoms, total disorganized symptoms, and site. | | | | | | |  |

| **Supplemental Table 4. Linear Regression Results of Covariates for Final Adjusted Models, *N*=113** | | | | | | |
| --- | --- | --- | --- | --- | --- | --- |
|  | **Self Esteem** | **Social Support Loss** | **QOL: Satisfaction with Life & Functioning** | **QOL: Satisfaction with Environment** | **QOL: Satisfaction Personal Relationships** | **QOL: Satisfaction with Health** |
|  | *B* (95% CI) | *B* (95% CI) | *B* (95% CI) | *B* (95% CI) | *B* (95% CI) | *B* (95% CI) |
| ***Panel A: Shame-Related Emotions*** | | | | | | |
| ***Final Subscale:*** | ***Symptom*** | ***Symptom*** | ***Symptom*** | ***--*** | ***--*** | ***Symptom*** |
| *N* | *92* | *99* | *96* | *96* | *96* | *96* |
| Age | **0.49^*^** | 0.04 | 0.00 | -- | -- | -0.05 |
|  | **(0.12, 0.87)** | (-0.01, 0.09) | (-0.04, 0.04) |  |  | (-0.11, 0.02) |
| Sex^a^ | 1.34 | -0.21 | 0.13 | -- | -- | -0.03 |
|  | (-1.07, 3.74) | (-0.56, 0.13) | (-0.12, 0.38) |  |  | (-0.44, 0.38) |
| Race/Ethnicity^b^ | 0.56 | -0.31 | -0.12 | -- | -- | -0.33 |
|  | (-2.11, 3.24) | (-0.70, 0.08) | (-0.41, 0.16) |  |  | (-0.80, 0.14) |
| Family History of Psychosis^c^ | 0.57 | 0.00 | -0.14 | -- | -- | 0.19 |
|  | (-1.80, 2.94) | (-0.34, 0.34) | (-0.39, 0.11) |  |  | (-0.23, 0.61) |
| Positive Symptoms | 0.07 | -0.02 | -0.02 | -- | -- | -0.06 |
|  | (-0.30, 0.44) | (-0.07, 0.04) | (-0.06, 0.02) |  |  | (-0.12, 0.01) |
| Negative Symptoms | **-0.30^**^** | 0.00 | **-0.04^***^** | -- | -- | -0.02 |
|  | **(-0.52, -0.08)** | (-0.03, 0.03) | **(-0.07, -0.02)** |  |  | (-0.06, 0.02) |
| Disorganized Symptoms | -0.05 | **0.07^*^** | 0.01 | -- | -- | 0.02 |
|  | (-0.54, 0.43) | **(0.00, 0.14)** | (-0.04, 0.07) |  |  | (-0.07, 0.10) |
| Site: Maine^d^ | -2.04 | 0.22 | -0.14 | -- | -- | -0.48 |
|  | (-5.00, 0.88) | (-0.21, 0.65) | (-0.44, 0.17) |  |  | (-0.99, 0.03) |
| Site: Columbia^e^ | 0.34 | -0.40 | 0.31 | -- | -- | 0.00 |
|  | (-3.48, 4.16) | (-0.95, 0.15) | (-0.09, 0.72) |  |  | (-0.67, 0.67) |
| ***Panel B: Secrecy*** | | | | | | |
| ***Final Subscale:*** | ***--*** | ***--*** | ***--*** | ***--*** | ***--*** | ***Symptom*** |
| *N* | *91* | *97* | *94* | *94* | *94* | *94* |
| Age | -- | -- | -- | -- | -- | **-0.07^*^** |
|  |  |  |  |  |  | **(-0.13, -0.00)** |
| Sex^a^ | -- | -- | -- | -- | -- | 0.13 |
|  |  |  |  |  |  | (-0.26, 0.52) |
| Race/Ethnicity^b^ | -- | -- | -- | -- | -- | -0.18 |
|  |  |  |  |  |  | (-0.65, 0.29) |
| Family History of Psychosis^c^ | -- | -- | -- | -- | -- | 0.29 |
|  |  |  |  |  |  | (-0.12, 0.70) |
| Positive Symptoms | -- | -- | -- | -- | -- | **-0.07^*^** |
|  |  |  |  |  |  | **(-0.13, -0.01)** |
| Negative Symptoms | -- | -- | -- | -- | -- | -0.04 |
|  |  |  |  |  |  | (-0.07, 0.00) |
| Disorganized Symptoms | -- | -- | -- | -- | -- | 0.02 |
|  |  |  |  |  |  | (-0.06, 0.10) |
| Site: Maine^c^ | -- | -- | -- | -- | -- | -0.07 |
|  |  |  |  |  |  | (-0.58, 0.43) |
| Site: Columbia^d^ | -- | -- | -- | -- | -- | 0.17 |
|  |  |  |  |  |  | (-0.49, 0.83) |
| ***Panel C: Discrimination*** | | | | | | |
| ***Final Subscale:*** | ***Labeling*** | ***Symptom*** | ***Symptom*** | ***Symptom*** | ***Symptom*** | ***Symptom*** |
| *N* | *91* | *98* | *95* | *95* | *95* | *95* |
| Age | 0.23 | 0.04 | 0.00 | -0.03 | **-0.12^***^** | -0.06 |
|  | (-0.17, 0.63) | (-0.01, 0.09) | (-0.04, 0.04) | (-0.07, 0.01) | **(-0.17, -0.06)** | (-0.13, 0.01) |
| Sex^a^ | **3.62^**^** | **-0.42^**^** | **0.26^*^** | 0.17 | -0.22 | 0.16 |
|  | **(1.13, 6.10)** | **(-0.73, -0.12)** | **(0.01, 0.50)** | (-0.09, 0.43) | (-0.56, 0.12) | (-0.23, 0.56) |
| Race/Ethnicity^b^ | 0.67 | **-0.42^*^** | -0.08 | -0.01 | 0.06 | -0.26 |
|  | (-2.22, 3.56) | **(-0.78, -0.06)** | (-0.38, 0.22) | (-0.32, 0.30) | (-0.35, 0.46) | (-0.74, 0.21) |
| Family History of Psychosis^c^ | 0.28 | -0.02 | -0.12 | **-0.39^**^** | 0.01 | 0.17 |
|  | (-2.30, 2.85) | (-0.34, 0.30) | (-0.38, 0.14) | **(-0.66, -0.11)** | (-0.35, 0.37) | (-0.25, 0.59) |
| Positive Symptoms | -0.03 | 0.01 | -0.03 | -0.04 | -0.01 | **-0.08^*^** |
|  | (-0.43, 0.36) | (-0.04, 0.06) | (-0.07, 0.01) | (-0.08, 0.00) | (-0.07, 0.04) | **(-0.15, -0.02)** |
| Negative Symptoms | **-0.40^**^** | 0.01 | **-0.05^***^** | **-0.04^**^** | -0.03 | -0.03 |
|  | **(-0.64, -0.17)** | (-0.02, 0.04) | **(-0.07, -0.02)** | **(-0.06, -0.01)** | (-0.06, 0.01) | (-0.07, 0.01) |
| Disorganized Symptoms | 0.20 | 0.01 | 0.04 | **0.07^*^** | -0.01 | 0.05 |
|  | (-0.32, 0.73) | (-0.05, 0.08) | (-0.01, 0.09) | **(0.01, 0.13)** | (-0.08, 0.07) | (-0.04, 0.14) |
| Site: Maine^c^ | -0.49 | -0.02 | 0.01 | 0.07 | -0.18 | -0.36 |
|  | (-3.53, 2.56) | (-0.41, 0.37) | (-0.30, 0.33) | (-0.26, 0.40) | (-0.61, 0.24) | (-0.86, 0.14) |
| Site: Columbia^d^ | -0.05 | -0.34 | 0.32 | -0.17 | 0.23 | 0.02 |
|  | (-4.26, 4.15) | (-0.85, 0.18) | (-0.10, 0.74) | (-0.61, 0.27) | (-0.34, 0.81) | (-0.66, 0.69) |
| *Note*. Betas are unstandardized. **^*^p<0.05, ^**^p<0.01, ^***^p<0.001**; ^a^Sex: Male vs. Female [Reference Group]; ^b^Race/Ethnicity: White non-Hispanic vs. All other races [Reference Group]; ^c^Family history of psychosis : Present vs. Absent [Reference Group];  ^d^Maine: Maine vs. Harvard site [Reference Group]; ^e^Columbia: Columbia vs. Harvard site [Reference Group]; double-dash (--) in unpopulated cells indicates that neither the labeling nor the symptom subscale moved to the Final Adjusted Model, therefore covariate parameter estimates are not shown. | | | | | | |

| **Supplemental Table 5. Regression Models of Stigma Domain and Psychosocial Variables: Bivariate Models Among All Individuals Meeting Criteria for CHR-p, *N*=150** | | | | | | |
| --- | --- | --- | --- | --- | --- | --- |
|  | **Self Esteem** | **Social Support Loss** | **QOL: Satisfaction with Life & Functioning** | **QOL: Satisfaction with Environment** | **QOL: Satisfaction Personal Relationships** | **QOL: Satisfaction with Health** |
|  | *B* (95% CI) | *B* (95% CI) | *B* (95% CI) | *B* (95% CI) | *B* (95% CI) | *B* (95% CI) |
| ***Panel A: Shame-Related Emotions*** | | | | | | |
| *N* | *113* | *121* | *118* | *118* | *118* | *118* |
| Labeling | **-0.79^**^** | **0.16^***^** | **-0.06^**^** | **-0.05^*^** | **-0.08^*^** | -0.03 |
|  | **(-1.26, -0.32)** | **(0.10, 0.21)** | **(-0.11, -0.02)** | **(-0.10, -0.00)** | **(-0.15, -0.02)** | (-0.10, 0.05) |
| Symptom | **-1.13^***^** | **0.16^***^** | **-0.11^***^** | **-0.06^*^** | **-0.08^*^** | **-0.11^**^** |
|  | **(-1.57, -0.70)** | **(0.11, 0.22)** | **(-0.16, -0.07)** | **(-0.11, -0.01)** | **(-0.15, -0.02)** | **(-0.18, -0.04)** |
| ***Panel B: Secrecy*** | | | | | | |
| *N* | *112* | *119* | *116* | *116* | *116* | *116* |
| Labeling | 0.20 | 0.05 | 0.05 | 0.04 | -0.08 | 0.09 |
|  | (-0.56, 0.96) | (-0.05, 0.15) | (-0.03, 0.12) | (-0.04, 0.11) | (-0.18, 0.06) | (-0.03, 0.20) |
| Symptom | 0.12 | 0.00 | 0.05 | 0.01 | -0.06 | **0.20^**^** |
|  | (-0.73, 0.98) | (-0.11, 0.12) | (-0.03, 0.14) | (-0.08, 0.10) | (-0.18, 0.06) | **(0.08, 0.33)** |
| ***Panel C: Discrimination*** | | | | | | |
| *N* | *111* | *119* | *116* | *116* | *116* | *116* |
| Labeling | **-0.27^*^** | 0.02 | **-0.02^*^** | -0.01 | -0.02 | -0.02 |
|  | **(-0.48, -0.05)** | (-0.01, 0.05) | **(-0.05, -0.00)** | (-0.03, 0.01) | (-0.05, 0.01) | (-0.05, 0.02) |
| Symptom | **-0.33^**^** | **0.09^***^** | **-0.05^***^** | **-0.04^***^** | **-0.05^**^** | **-0.05^**^** |
|  | **(-0.56, -0.09)** | **(0.06, 0.11)** | **(-0.07, -0.02)** | **(-0.07, -0.02)** | **(-0.08, -0.02)** | **(-0.09, -0.02)** |
| *Note.* Betas are unstandardized. Significance at **^*^p<0.05, ^**^p<0.01, ^***^p<0.001**. | | | | | | |

| **Supplemental Table 6. Linear Regression Models between Stigma Domain and Psychosocial Variables: Multivariable Models and Adjusted Models Among All Individuals Meeting Criteria for CHR-p, *N*=150** | | | | | | |
| --- | --- | --- | --- | --- | --- | --- |
|  | **Self Esteem** | **Social Support Loss** | **QOL: Satisfaction with Life & Functioning** | **QOL: Satisfaction with Environment** | **QOL: Satisfaction Personal Relationships** | **QOL: Satisfaction with Health** |
|  | *B* (95% CI) | *B* (95% CI) | *B* (95% CI) | *B* (95% CI) | *B* (95% CI) | *B* (95% CI) |
| ***Panel A: Shame-Related Emotions*** | | | | | | |
| *N* | *113* | *121* | *118* | *118* | *118* | *118* |
| **Multivariable Model** | | | | | | |
| Labeling | -0.11 | **0.09^*^** | 0.02 | -0.02 | -0.05 | 0.08 |
|  | (-0.68, 0.46) | **(0.01, 0.16)** | (-0.04, 0.08) | (-0.08, 0.04) | (-0.14, 0.04) | (-0.01, 0.17) |
| Symptom | **-1.06^***^** | **0.11^**^** | **-0.12^***^** | -0.05 | -0.05 | **-0.16^***^** |
|  | **(-1.63, -0.50)** | **(0.03, 0.18)** | **(-0.18, -0.07)** | (-0.11, 0.02) | (-0.14, 0.04) | **(-0.25, -0.07)** |
| **Final Adjusted Model** | | | | | | |
| ***Final Subscale:*** | ***Symptoms*** | ***Symptoms*** | ***Symptoms*** | ***--*** | ***--*** | ***Symptoms*** |
|  | **-1.28^***^** | **0.15^***^** | **-0.10^***^** | **--** | **--** | **-0.11^**^** |
|  | **(-1.71, -0.84)** | **(0.08, 0.21)** | **(-0.14, -0.06)** |  |  | **(-0.18, -0.04)** |
| ***Panel B: Secrecy*** | | | | | | |
| *N* | *112* | *119* | *116* | *116* | *116* | *116* |
| **Multivariable Model** | | | | | | |
| Labeling | 0.20 | 0.07 | 0.03 | 0.04 | -0.07 | 0.00 |
|  | (-0.68, 1.08) | (-0.05, 0.18) | (-0.06, 0.12) | (-0.05, 0.13) | (-0.19, 0.06) | (-0.13, 0.12) |
| Symptom | 0.01 | -0.04 | 0.04 | -0.01 | -0.02 | **0.20^**^** |
|  | (-0.98, 1.01) | (-0.17, 0.09) | (-0.06, 0.14) | (-0.12, 0.09) | (-0.16, 0.12) | **(0.06, 0.35)** |
| **Final Adjusted Model** | | | | | | |
| ***Final Subscale:*** | ***--*** | ***--*** | ***--*** | ***--*** | ***--*** | ***Symptoms*** |
|  | **--** | **--** | **--** | **--** | **--** | **0.17^**^** |
|  |  |  |  |  |  | **(0.04, 0.29)** |
| ***Panel C: Discrimination*** | | | | | | |
| *N* | *111* | *119* | *116* | *116* | *116* | *116* |
| **Multivariable Model** | | | | | | |
| Labeling | -0.15 | -0.03 | 0.00 | 0.01 | 0.01 | 0.01 |
|  | (-0.40, 0.11) | (-0.06, 0.00) | (-0.03, 0.02) | (-0.01, 0.04) | (-0.02, 0.05) | (-0.03, 0.05) |
| Symptom | -0.24 | **0.10^***^** | **-0.05^**^** | **-0.05^***^** | **-0.06^**^** | **-0.06^**^** |
|  | (-0.52, 0.04) | **(0.07, 0.13)** | **(-0.07, -0.02)** | **(-0.08, -0.02)** | **(-0.09, -0.02)** | **(-0.10, -0.02)** |
| **Final Adjusted Model** | | | | | | |
| ***Final Subscale:*** | ***--*** | ***Symptoms*** | ***Symptoms*** | ***Symptoms*** | ***Symptoms*** | ***Symptoms*** |
|  | **--** | **0.09^***^** | **-0.03^**^** | **-0.04^**^** | **-0.04^*^** | **-0.04^*^** |
|  |  | **(0.06, 0.12)** | **(-0.05, -0.01)** | **(-0.06, -0.02)** | **(-0.07, -0.01)** | **(-0.07, -0.00)** |
| *Note.* Betas are unstandardized. Significance at **^*^p<0.05, ^**^p<0.01, ^***^p<0.001**. Final Adjusted Models adjust for: age, sex, race, family history of psychosis, total positive symptoms, total negative symptoms, total disorganized symptoms, and site; double-dash (--) in unpopulated cells indicates that neither the labeling nor the symptom subscale moved to the Final Adjusted Model. | | | | | | |

| **Supplemental Table 7. Regression Models of Stigma Domain and Psychosocial Variables: Bivariate Models Among Subsample of Individuals Reporting Awareness of Psychosis-risk Who Were Also Conveyed a CHR-p Designation, N=89** | | | | | | |
| --- | --- | --- | --- | --- | --- | --- |
|  | **Self Esteem** | **Social Support Loss** | **QOL: Satisfaction with Life & Functioning** | **QOL: Satisfaction with Environment** | **QOL: Satisfaction Personal Relationships** | **QOL: Satisfaction with Health** |
|  | *B* (95% CI) | *B* (95% CI) | *B* (95% CI) | *B* (95% CI) | *B* (95% CI) | *B* (95% CI) |
| ***Panel A: Shame-Related Emotions*** | | | | | |  |
| *N* | *71* | *77* | *74* | *74* | *74* | *74* |
| Labeling | **-0.77^*^** | **0.19^***^** | **-0.07^*^** | -0.06 | **-0.09^*^** | -0.03 |
|  | **(-1.35, -0.19)** | **(0.12, 0.25)** | **(-0.12, -0.01)** | (-0.12, 0.00) | **(-0.17, -0.01)** | (-0.12, 0.06) |
| Symptom | **-1.09^***^** | **0.21^***^** | **-0.11^***^** | -0.04 | **-0.11^*^** | **-0.11^*^** |
|  | **(-1.66, -0.52)** | **(0.15, 0.28)** | **(-0.17, -0.06)** | (-0.10, 0.02) | **(-0.19, -0.03)** | **(-0.20, -0.02)** |
| ***Panel B: Secrecy*** | | | | | |  |
| *N* | *71* | *76* | *73* | *73* | *73* | *73* |
| Labeling | 0.19 | 0.08 | 0.04 | 0.02 | -0.08 | 0.11 |
|  | (-0.75, 1.13) | (-0.05, 0.20) | (-0.05, 0.13) | (-0.07, 0.12) | (-0.21, 0.05) | (-0.03, 0.26) |
| Symptom | 0.17 | 0.02 | 0.06 | 0.01 | -0.01 | **0.25^**^** |
|  | (-0.85, 1.20) | (-0.12, 0.16) | (-0.04, 0.16) | (-0.10, 0.12) | (-0.16, 0.13) | **(0.10, 0.40)** |
| ***Panel C: Discrimination*** | | | | | |  |
| *N* | *70* | *76* | *73* | *73* | *73* | *73* |
| Labeling | **-0.45^**^** | 0.03 | **-0.03^*^** | -0.01 | -0.03 | -0.03 |
|  | **(-0.72, -0.19)** | (-0.01, 0.06) | **(-0.06, -0.00)** | (-0.04, 0.02) | (-0.07, 0.01) | (-0.07, 0.01) |
| Symptom | **-0.37^*^** | **0.10^***^** | **-0.04^*^** | **-0.05^***^** | **-0.08^***^** | **-0.06^**^** |
|  | **(-0.67, -0.07)** | **(0.07, 0.13)** | **(-0.07, -0.01)** | **(-0.08, -0.02)** | **(-0.11, -0.04)** | **(-0.11, -0.02)** |
| *Note.* Betas are unstandardized. Significance at **^*^p<0.05, ^**^p<0.01, ^***^p<0.001**. | | | | | |  |

| **Supplemental Table 8. Linear Regression Models between Stigma Domain and Psychosocial Variables: Multivariable Models and Adjusted Models Among Subsample of Individuals Reporting Awareness of Psychosis-risk Who Were Also Conveyed a CHR-p Designation, N=89** | | | | | | |
| --- | --- | --- | --- | --- | --- | --- |
|  | **Self Esteem** | **Social Support Loss** | **QOL: Satisfaction with Life & Functioning** | **QOL: Satisfaction with Environment** | **QOL: Satisfaction Personal Relationships** | **QOL: Satisfaction with Health** |
|  | *B* (95% CI) | *B* (95% CI) | *B* (95% CI) | *B* (95% CI) | *B* (95% CI) | *B* (95% CI) |
| ***Panel A: Shame-Related Emotions*** | | | | | |  |
| *N* | *71* | *77* | *74* | *74* | *74* | *74* |
| **Multivariable Model** | | | | | |  |
| Labeling | -0.04 | 0.08 | 0.02 | -0.06 | -0.02 | 0.09 |
|  | (-0.83, 0.75) | (-0.01, 0.17) | (-0.05, 0.10) | (-0.15, 0.03) | (-0.14, 0.10) | (-0.04, 0.21) |
| Symptom | **-1.06^*^** | **0.16^**^** | **-0.13^**^** | 0.00 | -0.09 | **-0.17^*^** |
|  | **(-1.87, -0.25)** | **(0.06, 0.25)** | **(-0.21, -0.05)** | (-0.09, 0.10) | (-0.22, 0.03) | **(-0.30, -0.04)** |
| **Final Adjusted Model** | | | | | |  |
| ***Final Subscale:*** | ***Symptom*** | ***Symptom*** | ***Symptom*** | -- | -- | ***Symptoms*** |
|  | **-1.25^***^** | **0.21^***^** | **-0.11^***^** | -- | -- | -0.10 |
|  | **(-1.77, -0.73)** | **(0.14, 0.28)** | **(-0.16, -0.06)** |  |  | (-0.20, 0.00) |
| ***Panel B: Secrecy*** | | | | | |  |
| *N* | *71* | *76* | *73* | *73* | *73* | *73* |
| **Multivariable Model** | | | | | |  |
| Labeling | 0.15 | 0.10 | 0.01 | 0.03 | -0.11 | -0.01 |
|  | (-0.97, 1.28) | (-0.05, 0.24) | (-0.09, 0.12) | (-0.09, 0.15) | (-0.26, 0.05) | (-0.18, 0.15) |
| Symptom | 0.08 | -0.04 | 0.05 | -0.01 | 0.05 | **0.26^**^** |
|  | (-1.14, 1.31) | (-0.20, 0.12) | (-0.07, 0.17) | (-0.14, 0.12) | (-0.13, 0.22) | **(0.08, 0.44)** |
| **Final Adjusted Model** | | | | | |  |
| ***Final Subscale:*** | -- | -- | -- | -- | -- | ***Symptoms*** |
|  | -- | -- | -- | -- | -- | **0.27^**^** |
|  |  |  |  |  |  | **(0.11, 0.43)** |
| ***Panel C: Discrimination*** | | | | | |  |
| *N* | *70* | *76* | *73* | *73* | *73* | *73* |
| **Multivariable Model** | | | | | |  |
| Labeling | **-0.38^*^** | -0.03 | -0.02 | 0.02 | 0.01 | 0.00 |
|  | **(-0.70, -0.07)** | (-0.06, 0.01) | (-0.05, 0.02) | (-0.01, 0.05) | (-0.03, 0.05) | (-0.05, 0.04) |
| Symptom | -0.14 | **0.11^***^** | **-0.03^*^** | **-0.07^***^** | **-0.08^***^** | **-0.06^*^** |
|  | (-0.49, 0.20) | **(0.08, 0.15)** | **(-0.07, -0.00)** | **(-0.10, -0.03)** | **(-0.13, -0.04)** | **(-0.11, -0.01)** |
| **Final Adjusted Model** | | | | | |  |
| ***Final Subscale:*** | ***Labeling*** | ***Symptoms*** | ***Symptoms*** | ***Symptoms*** | ***Symptoms*** | ***Symptoms*** |
|  | **-0.38^*^** | **0.10^***^** | -0.03 | **-0.05^**^** | **-0.05^*^** | -0.04 |
|  | **(-0.67, -0.09)** | **(0.07, 0.14)** | (-0.06, 0.00) | **(-0.08, -0.02)** | **(-0.09, -0.01)** | (-0.09, 0.01) |
| *Note.* Betas are unstandardized. Significance at **^*^p<0.05, ^**^p<0.01, ^***^p<0.001**. Final Adjusted Models adjust for: age, sex, race, family history of psychosis, total positive symptoms, total negative symptoms, total disorganized symptoms, and site; double-dash (--) in unpopulated cells indicates that neither the labeling nor the symptom subscale moved forward to the Final Adjusted Model. | | | | | | |

| **Supplemental Table 9.** Stigma and Race/Ethnicity^a^ Interactions for Final Adjusted Linear Regression Models, *N*=113 | | | | | | |
| --- | --- | --- | --- | --- | --- | --- |
|  | **Self Esteem** | **Social Support Loss** | **QOL: Satisfaction with Life & Functioning** | **QOL: Satisfaction with Environment** | **QOL: Satisfaction Personal Relationships** | **QOL: Satisfaction with Health** |
|  | *B* (95% CI) | *B* (95% CI) | *B* (95% CI) | *B* (95% CI) | *B* (95% CI) | *B* (95% CI) |
| ***Panel A: Shame-Related Emotions*** | | | | | |  |
| *N* | *92* | *99* | *96* | *96* | *96* | *96* |
| ***Final Subscale:*** | ***Symptom*** | ***Symptom*** | ***Symptom*** | -- | -- | ***Symptoms*** |
| *Stigma Subscale* | **-1.02^**^** | **0.20^***^** | **-0.09^*^** | -- | -- | -0.06 |
|  | **(-1.77, -0.27)** | **(0.09, 0.31)** | **(-0.16, -0.01)** |  |  | (-0.18, 0.07) |
| *Race/Ethnicity* | 2.86 | 0.21 | 0.04 | -- | -- | 0.47 |
|  | (-4.66, 10.38) | (-0.91, 1.32) | (-0.75, 0.83) |  |  | (-0.83, 1.76) |
| *Stigma Subscale * Race/Ethnicity* | -0.32 | -0.07 | -0.02 | -- | -- | -0.11 |
|  | (-1.31, 0.66) | (-0.22, 0.07) | (-0.13, 0.08) |  |  | (-0.28, 0.06) |
| ***Panel B: Secrecy*** | | | | | |  |
| *N* | *91* | *97* | *94* | *94* | *94* | *94* |
| ***Final Subscale:*** | -- | -- | -- | -- | -- | ***Symptoms*** |
| *Stigma Subscale* | -- | -- | -- | -- | -- | **0.22^*^** |
|  |  |  |  |  |  | **(0.01, 0.43)** |
| *Race/Ethnicity* | -- | -- | -- | -- | -- | -0.22 |
|  |  |  |  |  |  | (-0.98, 0.54) |
| *Stigma Subscale * Race/Ethnicity* | -- | -- | -- | -- | -- | 0.02 |
|  |  |  |  |  |  | (-0.27, 0.30) |
| ***Panel C: Discrimination*** | | | | | |  |
| *N* | *91* | *98* | *95* | *95* | *95* | *95* |
| ***Final Subscale:*** | ***Labeling*** | ***Symptoms*** | ***Symptoms*** | ***Symptoms*** | ***Symptoms*** | ***Symptoms*** |
| *Stigma Subscale* | -0.37 | **0.07^*^** | -0.02 | **-0.09^***^** | -0.01 | -0.03 |
|  | (-0.83, 0.09) | **(0.02, 0.12)** | (-0.07, 0.02) | **(-0.14, -0.04)** | (-0.07, 0.05) | (-0.10, 0.05) |
| *Race/Ethnicity* | 0.60 | **-0.90^*^** | 0.16 | **-0.71^*^** | 0.39 | -0.10 |
|  | (-5.10, 6.31) | **(-1.68, -0.12)** | (-0.47, 0.78) | **(-1.34, -0.08)** | (-0.47, 1.24) | (-1.11, 0.90) |
| *Stigma Subscale * Race/Ethnicity* | 0.01 | 0.05 | -0.02 | **0.07^*^** | -0.03 | -0.02 |
|  | (-0.54, 0.55) | (-0.02, 0.12) | (-0.08, 0.03) | **(0.02, 0.13)** | (-0.11, 0.04) | (-0.11, 0.07) |
| *Note.* Betas are unstandardized. Significance at **^*^p<0.05, ^**^p<0.01, ^***^p<0.001**. Final Adjusted Models adjust for: age, sex, race, family history of psychosis, total positive symptoms, total negative symptoms, total disorganized symptoms, and site; double-dash (--) in unpopulated cells indicates that neither the labeling nor the symptom subscale moved forward to the Final Adjusted Model. ^a^Race/Ethnicity: White non-Hispanic vs. All other races [Reference Group]  Brief Discussion: Interaction analyses reveal a stronger association between symptom-related discrimination and satisfaction with environment among Non-White and/or Hispanic respondents compared with White/Non-Hispanic participants (interaction term *B*=0.07; 95% CI [0.02, 0.13], R^2^=0.26 *p*<.05) | | | | | | |

| **Supplemental Table 10.** Stigma and Self-reported Family History of Psychosis^a^ Interactions for Final Adjusted Linear Regression Models, *N*=113 | | | | | | |
| --- | --- | --- | --- | --- | --- | --- |
|  | **Self Esteem** | **Social Support Loss** | **QOL: Satisfaction with Life & Functioning** | **QOL: Satisfaction with Environment** | **QOL: Satisfaction Personal Relationships** | **QOL: Satisfaction with Health** |
|  | *B* (95% CI) | *B* (95% CI) | *B* (95% CI) | *B* (95% CI) | *B* (95% CI) | *B* (95% CI) |
| ***Panel A: Shame-Related Emotions*** | | | | | |  |
| *N* | *92* | *99* | *96* | -- | -- | *96* |
| ***Final Subscale:*** | ***Symptom*** | ***Symptom*** | ***Symptom*** | -- | -- | ***Symptoms*** |
| *Stigma Subscale* | **-1.37^***^** | **0.12^*^** | **-0.15^***^** | -- | -- | **-0.19^***^** |
|  | **(-2.01, -0.74)** | **(0.03, 0.21)** | **(-0.21, -0.08)** |  |  | **(-0.30, -0.09)** |
| *Family History of Psychosis* | -2.77 | -0.84 | **-1.07^**^** | -- | -- | **-1.28^*^** |
|  | (-10.16, 4.62) | (-1.90, 0.22) | **(-1.81, -0.33)** |  |  | **(-2.50, -0.05)** |
| *Stigma Subscale * Family History* | 0.49 | 0.12 | **0.13^**^** | -- | -- | **0.21^*^** |
|  | (-0.53, 1.50) | (-0.02, 0.26) | **(0.03, 0.23)** |  |  | **(0.04, 0.38)** |
| ***Panel B: Secrecy*** | | | | | |  |
| *N* | -- | -- | -- | -- | -- | *94* |
| ***Final Subscale:*** | -- | -- | -- | -- | -- | ***Symptoms*** |
| *Stigma Subscale* | -- | -- | -- | -- | -- | 0.18 |
|  |  |  |  |  |  | (-0.00, 0.35) |
| *Family History of Psychosis* | -- | -- | -- | -- | -- | 0.00 |
|  |  |  |  |  |  | (-0.68, 0.68) |
| *Stigma Subscale * Family History* | -- | -- | -- | -- | -- | 0.16 |
|  |  |  |  |  |  | (-0.14, 0.45) |
| ***Panel C: Discrimination*** | | | | | |  |
| *N* | *91* | *98* | *95* | *95* | *95* | *95* |
| ***Final Subscale:*** | ***Labeling*** | ***Symptoms*** | ***Symptoms*** | ***Symptoms*** | ***Symptoms*** | ***Symptoms*** |
| *Stigma Subscale* | **-0.39^*^** | **0.09^***^** | **-0.05^*^** | **-0.06^**^** | -0.03 | -0.05 |
|  | **(-0.71, -0.08)** | **(0.05, 0.13)** | **(-0.08, -0.01)** | **(-0.10, -0.02)** | (-0.08, 0.02) | (-0.11, 0.00) |
| *Family History of Psychosis* | -0.46 | -0.23 | -0.35 | **-0.79^*^** | 0.10 | -0.27 |
|  | (-5.80, 4.88) | (-1.01, 0.55) | (-0.97, 0.26) | **(-1.43, -0.16)** | (-0.75, 0.95) | (-1.26, 0.72) |
| *Stigma Subscale * Family History* | 0.08 | 0.02 | 0.02 | 0.04 | -0.01 | 0.04 |
|  | (-0.41, 0.56) | (-0.05, 0.09) | (-0.03, 0.08) | (-0.02, 0.09) | (-0.08, 0.06) | (-0.04, 0.13) |
| *Note.* Betas are unstandardized. Significance at **^*^p<0.05, ^**^p<0.01, ^***^p<0.001**. Final Adjusted Models adjust for: age, sex, race, family history of psychosis, total positive symptoms, total negative symptoms, total disorganized symptoms, and site; double-dash (--) in unpopulated cells indicates that neither the labeling nor the symptom subscale moved forward to the Final Adjusted Model. ^a^Family history of psychosis: Present vs. Absent [Reference Group]  Brief Discussion: For those without family history of psychosis (compared to those with family history), symptom shame is more strongly associated with satisfaction with life and functioning (interaction term *B*=0.13; 95% CI [0.03, 0.23], R^2^=0.30, *p*<.01) and satisfaction with health (interaction term *B*=0.21; 95% CI [0.04, 0.38], R^2^=0.16, *p*<.05) | | | | | | |


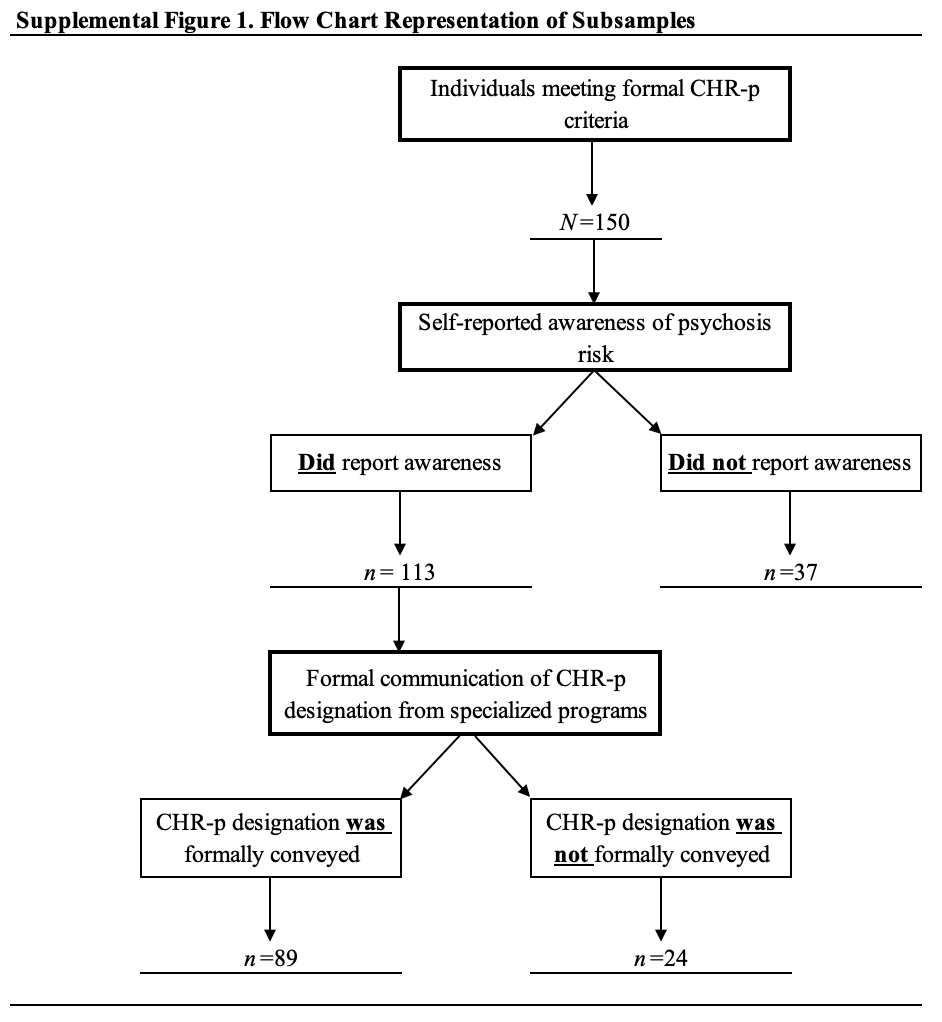
­­­­­­­­
